# IgM triplet in neonatal diagnosis by immunoblotting and its potential use as a diagnostic marker for congenital toxoplasmosis[Fn FN1]

**DOI:** 10.1051/parasite/2023020

**Published:** 2023-06-02

**Authors:** Lucie Peyclit, Odile Villard, Luc Paris, Hélène Fricker-Hidalgo, Sandrine Houzé, Bernard Cimon, Anne-Sophie Deleplancque, Céline Tournus, Hervé Pelloux, Isabelle Villena, Christelle Pomares, Coralie L’Ollivier

**Affiliations:** 1 IHU Méditerranée Infection 13005 Marseille France; 2 Laboratoire de Parasitologie et Mycologie Médicale, CNR de la Toxoplasmose, Les Hôpitaux Universitaires de Strasbourg 67091 Strasbourg France; 3 Institut de Parasitologie et Pathologie Tropicale, UR7292 Dynamique des interactions hôte pathogène, Fédération de Médecine Translationnelle, Université de Strasbourg 67091 Strasbourg France; 4 AP-HP, Hôpital Pitié-Salpêtrière, Service de Parasitologie Mycologie 75013 Paris France; 5 Service de Parasitologie Mycologie, CHU Grenoble Alpes et Université Grenoble Alpes 38700 Grenoble France; 6 Service de Parasitologie Mycologie, Hôpital Bichat-Claude Bernard, AP-HP, 75018 Paris, et UMR 261 Merit Université Paris Cité 75018 Paris France; 7 Service de Parasitologie Mycologie, CHU Angers 49100 Angers France; 8 Laboratoire de Parasitologie Mycologie, CHU Lille 59037 Lille France; 9 Laboratoire de Microbiologie, Centre Hospitalier de Saint-Denis 93200 Saint-Denis France; 10 Service de Parasitologie Mycologie, EA 7510 Université Reims-Champagne Ardenne, Centre National de Référence de la Toxoplasmose, Centre Hospitalier Universitaire (CHU) Reims 51092 Reims France; 11 Service de Parasitologie Mycologie, Université de la Côte d’Azur, C3M INSERM 1065, CHU Nice 06204 Nice France; 12 Aix Marseille Univ, IRD, AP-HM, SSA, VITROME 13005 Marseille France

**Keywords:** *Toxoplasma gondii*, Congenital toxoplasmosis, Neonatal diagnosis, Immunoblotting, IgM triplet

## Abstract

Primary infection during pregnancy by the protozoan *Toxoplasma gondii* can be worrisome because transmission to the fetus may lead to congenital toxoplasmosis (CT). Neonatal diagnosis is usually performed by serological profile comparison of the mother and newborn. As previously reported in 2012 by C. L’Ollivier et al., three IgM bands at 75, 90 and 100 kDa called the “IgM triplet” has caught our attention and seems to be pathognomonic of CT. This retrospective multicenter study involved nine reference laboratories included in the French National Reference Center for Toxoplasmosis network and concerned determining the specificity and sensitivity of this IgM triplet. On this basis, we were able to propose a new read of the comparison of IgG and IgM immunoblot profiles of mother and infant to increase the sensitivity of this diagnostic marker. The effect of the trimester of pregnancy at the time of infection, but also of maternal treatment with pyrimethamine/sulfadiazine/folinic acid on the presence of this IgM triplet in the infant, could be studied. The presence of the triplet appears pathognomonic for the diagnosis of CT, and it increased the sensitivity of the immunoblot assay from 55.04% to 72.48%. As a result, it would be wise to enhance conventional immunoblot reading by adding the presence of the three IgM bands in the infant pattern for neonatal diagnosis of CT.

## Abstract (fr)

La primo-infection pendant la grossesse par le protozoaire *Toxoplasma gondii* peut se révéler préoccupante car la transmission au fœtus peut conduire à une toxoplasmose congénitale (TC). Un diagnostic néonatal est généralement réalisé par comparaison des profils sérologiques de la mère et du nouveau-né. Comme précédemment rapporté en 2012 par C. L’Ollivier *et al.*, l’association de trois bandes d’IgM à 75, 90, et 100 kDa appelée la « triplette IgM » a retenu notre attention et semble être pathognomonique de la TC. Cette étude rétrospective multicentrique impliquant neuf laboratoires de référence inclus dans le réseau du Centre National de Référence pour la Toxoplasmose a permis de déterminer la spécificité et la sensibilité de cette triplette IgM. Ainsi, cela a permis de proposer une nouvelle lecture de la comparaison des profils d’immunoblot IgG et IgM de la mère et du nourrisson pour augmenter la sensibilité de ce marqueur diagnostique. L’effet du trimestre de la grossesse au moment de l’infection mais aussi du traitement maternel par pyriméthamine/sulfadiazine/acide folinique sur la présence de la triplette IgM chez l’enfant a pu être analysé. La présence de cette triplette semble pathognomonique pour le diagnostic de TC et elle permet d’augmenter la sensibilité du test immunoblot de 55,04 % à 72,48 %. Ainsi, il pourrait être judicieux d’améliorer la lecture conventionnelle de l’immunoblot en ajoutant la présence des trois bandes IgM dans le schéma du nourrisson pour le diagnostic néonatal de TC.

## Introduction

The parasite *Toxoplasma gondii* is an intracellular protozoan that infects many people worldwide. While most human infections are asymptomatic, this parasite leads to significant harm in newborns and immunocompromized patients [4]. Primary infection with *T. gondii* during pregnancy raises concerns about the possible fetal infection called congenital toxoplasmosis (CT). The severity is variable and can have severe consequences for the fetus, such as miscarriage and severe neurologic or ocular lesions [7]. Therefore, diagnosis should be made as early as possible to start treatment aimed at preventing mother-to-child transmission and to minimize clinical sequelae in already infected offspring [10]. The laboratory diagnosis of CT can be made by detection of *T. gondii* DNA by polymerase chain reaction (PCR) in prenatal amniotic fluid or in postnatal amniotic fluid or umbilical cord/newborn blood, or/and by detection of neosynthesized IgG, IgM or IgA in the newborn, or/and by persistence of IgG within 12 months of life [8, 10]. The French national program for CT diagnosis comprises detection of neosynthesized antibodies by the newborn based on serological tests, such as testing for specific IgM and/or IgA using immunoanalysis, IgG kinetics during the follow-up by immunoanalysis, and detection of supplemental IgG and/or IgM anti-Toxoplasma in the newborn using immunoblot. The goal of comaparing IgG and IgM mother and infant serological profiles using immunoblot (IB) is to highlight a different pattern of IgG or IgM reactivity between the mother and her infant at birth. This test has provided significant advances in the early diagnosis of CT [9, 11, 12]. Previously, we demonstrated that the consideration of high molecular weight bands significantly improved the sensitivity of the test, without yielding false positive results. Particularly, three IgM bands associated at 75, 90 and 100 kDa called the “IgM triplet” caught our attention and seems to be pathognomonic of CT [5].

The aim of the present study was to assess the positive diagnostic value of the “IgM triplet” in establishing the earliest diagnosis of CT. This makes it possible to propose a new read of the comparison of IgG and IgM immunoblot profiles of mother and infant to increase the sensitivity of this diagnostic marker.

## Materials and methods

### Ethics

The study was conducted according to the guidelines of the Declaration of Helsinki and approved by the Ethics Committee of the Assistance Publique des Hôpitaux de Marseille (APHM) (protocol code 2019-73 on May 29, 2019).

### Collection of data

This retrospective multicenter study involved nine reference laboratories included in France’s National Reference Center for Toxoplasmosis network (NRCT) (Angers, Grenoble, Lille, Marseille, Nice, Paris Bichat, Paris Pitié Salpetrière, Reims and Strasbourg).

**Figure 1:**
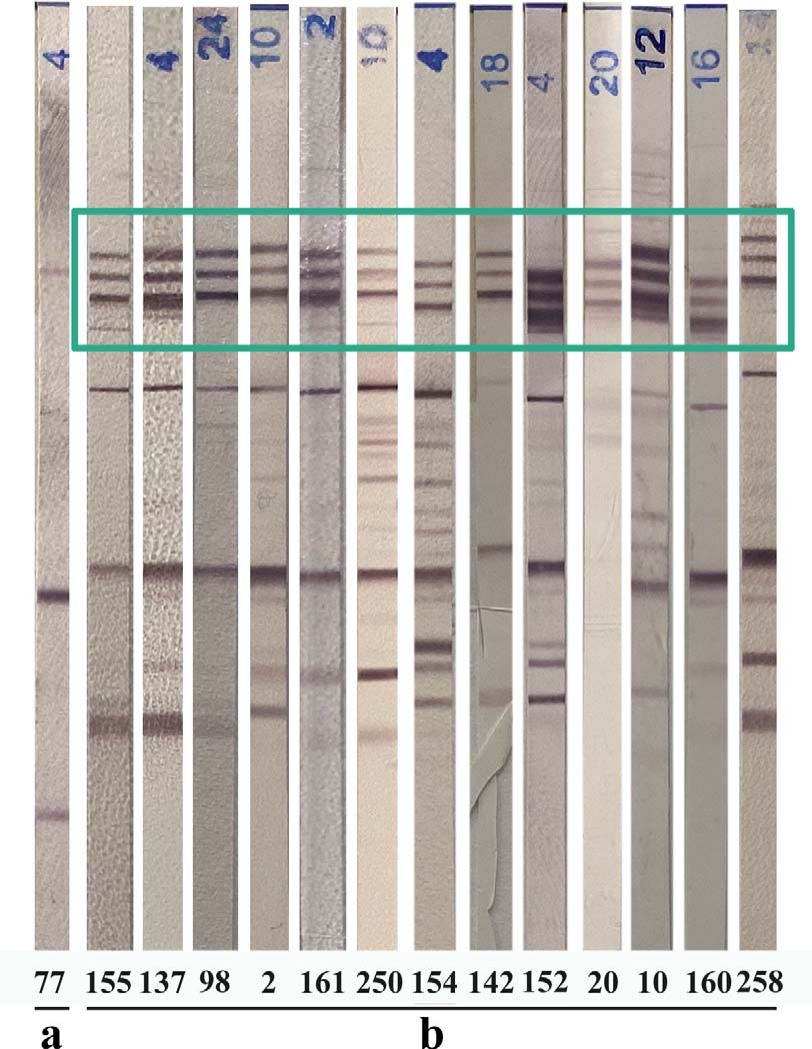
Example of immunoblot (LDBIO Diagnostics, Lyon, France) profiles of infected newborns showing the three IgM bands associated at 75, 90 and 100 kDa. a: pattern without IgM triplet; b: multiple patterns with IgM triplet (green box). The numbers refer to the patients mentioned in Table 1. All immunoblots were originated from different rainbow patterns.

From 2006 to 2020, all mothers who acquired maternal *T. gondii* infection during pregnancy and for whom prenatal diagnosis and/or infant postnatal follow-up were performed to prove (*n* = 258) or formally exclude (*n* = 237) congenital infection were included in this study. The practical approaches to CT diagnosis had been based on the recommendations of the NRCT [14]. IgM and IgG mother and child immunoblot pair profiles using cord blood or peripheral blood sampled at three days of life were retrospectively read by an operator from each center. A standard serum showing the triplet was sent to each center in order to have a standard showing the three specific bands. The reading was done by each center with the Marseille center as proofreading expert in the event of uncertainty. The presence of the three IgM bands associated at 75, 90 and 100 kDa was recorded (Fig. 1). We carefully noted the presence or absence of the IgM triplet in each mother and children strip. Other informative data were collected such as: maternal infection date, PCR results on amniotic fluid, the results of IgG/IgM mother-infant immunologic profiles (conventional reading according to manufacturer’s instructions) (Toxoplasma WB IgG-IgM, LDBIO Diagnostics, Lyon, France) and the presence or absence of IgM (Toxo-ISAGA, bioMérieux, and/or Platelia Toxo, BioRad) in cord blood or peripheral blood at the same time as the mother-infant immunoblot.

### Assessment of the analytical performance of the IgM triplet

First, the specificity of the IgM triplet was evaluated in the no congenital toxoplasmosis group (NTC group), corresponding to the group of toxoplasmosis-free children from per-partum infected mothers (*n* = 237). All immunoblot pair profiles were retrospectively reviewed to note the presence or absence of the IgM triplet on the mother and infant patterns, respectively.

**Figure 2:**
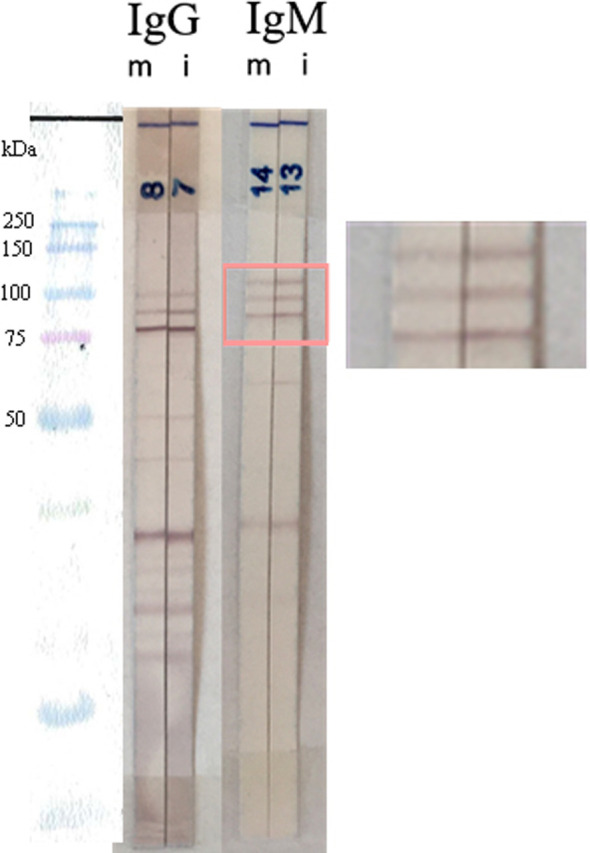
Example of an IgM and IgG mother and child immunoblot pair profile with identical profiles and the infant’s IgM triplet (pink box). The child was proved to be infected subsequently during follow-up. m, mother; i, infant.

Second, the sensitivity of the IgM triplet was evaluated in the congenital toxoplasmosis group (CT group) (*n* = 258). All immunoblot pair profiles were retrospectively reviewed to note the results of the conventional reading (i.e., the presence or absence of immunoblot bands in the newborn’s serum and not found in the maternal serum, indicating neosynthesized IgG and/or IgM) and to record the presence or absence of the IgM triplet on the mother and infant profiles, respectively. The sensitivity of the immunoblot assay in the diagnosis of CT was determined by adding the number of cases with different immunoblot profiles and the number of cases with a non-different immunoblot profile associated with the IgM triplet in the infant profile (Fig. 2 and Table 1).

**Table 1:** General data regarding interpretation of the IgM triplet.

CT group (*n* = 258)	Conventional reading IgG and IgM profiles	Conventional reading + presence of the infant’s IgM triplet
Different pattern	142	187
No supplementary bands	116	71

### Other parameters related to the presence of the infant IgM triplet

The presence and absence of the IgM triplet was analyzed according to the trimester of pregnancy at the time of infection and positive parasite DNA detection by quantitative PCR (qPCR) on amniotic fluid when available.

### Statistical analysis

Statistical analysis was performed using PRISM 5.0 and STATA 14.2. Detection rates of the IgM triplet in terms of the trimester of pregnancy at the time of infection and the positivity of the qPCR on amniotic fluid were compared across methods using a Chi^2^ test and a Clopper–Pearson test.

## Results

### Specificity of the IgM triplet

In the NTC group (*n* = 237), the IgM triplet was not found in any of the infant patterns. The presence of the IgM triplet in the mother’s profile appeared in 42 cases and was therefore not used for CT diagnosis. In addition, the specificity of the infant IgM triplet was 100%.

### Sensitivities of the infant IgM triplet

In the CT group (*n* = 258), the presence of neosynthesized IgM and/or IgG was detected in 142 infected newborns. A different immunologic profile from that of the mother was found in 122 and 78 newborns for the IgM and IgG patterns, respectively. The IgM triplet was detected in 140 newborns, 45 of whom had an identical IgG and IgM immunologic profile. The IgM triplet was detected in both mother and infant in 84 mother-child pairs. Taken together, the different immunologic profiles plus the infant IgM triplet led to detection of 187 CT cases in the newborns (Table 1 and Supplementary Table S1). Supplementary Table S1 provides detailed information for each newborn with CT, including the trimester of maternal infection, results of amniotic fluid qPCR, and the presence of the IgM triplet in both mother and infant profiles. The table also includes interpretations of conventional reading of IgG and IgM profiles, and conventional reading plus inclusion of the IgM triplet, and qualitative results of Platelia or ISAGA IgM. Finally, the sensitivity of the conventional reading of the mother–infant immunoblot profile, i.e., considering any supplementary well-defined band in infant serum, was 55.0%, whereas the association of the conventional reading plus the presence of the infant IgM triplet increased the sensitivity to 72.5% (Table 1).

### Effect of pregnancy trimester at the time of infection on the IgM triplet

In all CT cases, the trimester of maternal infection was the first trimester in 16/258 cases (6.2%), second in 74/258 cases (28.7%), third in 133/258 cases (51.5%), and unknown in 35/258 cases (13.6%). Depending on the knowledge of the trimester of infection, the percentages of the presence of the infant IgM triplet or its absence were: for the first trimester 43.8% (7/16) versus 56.2% (9/16), the second 37.8% (28/74) versus 62.2% (46/74), the third 67.7% (90/133) versus 32.3% (43/133), and when unknown 42.9% (15/35) versus 57.1% (20/35). The trimester of contamination had a significant influence on the occurrence of the IgM triplet (*p* < 0.0001) (Chi^2^ test between second trimester group and third trimester group). The sample size for the first trimester was too limited. The proportion of IgM triplets for contamination of the third trimester of pregnancy [67.7%; 95%CI: 59.01–75.51] is significantly higher than in the second trimester [37.8%; 95%CI: 26.8–49.8].

### Presence of the infant IgM triplet and maternal treatment with pyrimethamine/sulfadiazine/folinic acid (PS)

In the CT group, the amniotic fluid qPCR test results were positive, negative, not performed and with missing data in 109, 13, 98 and 38 cases, respectively. A positive qPCR result on amniotic fluid triggered treatment with PS. The IgM triplet was observed in 50 cases among 109 cases with positive amniotic fluid qPCR, in three cases when the amniotic fluid qPCR was negative (13 cases), in 56 and 30 when qPCR has not been performed (98 cases) or data were missing (38 cases), respectively. Sampling in each category was too small to be compared statistically.

## Discussion

Diagnosis of CT is based on biological tests performed during the prenatal and postnatal periods and mainly on serological tests in the neonatal period. The goal is to initiate treatment of the infected offspring as soon as possible to minimize clinical sequelae. It is recognized that comparing mother and infant IgG and IgM immunoblot profiles enables early neonatal diagnosis [13, 14]. To identify other diagnostic tests for early diagnosis of toxoplasma infection in newborns at risk of congenital toxoplasmosis, other approaches have been explored, such as evaluating specific T cell immunity to *T. gondii* antigens through measurement of lymphocyte proliferation and interferon-gamma production [2, 3]. Other authors have developed a multiplexed serology assay for detection of *T. gondii* IgG and IgM, rubella IgG, and CMV IgG, in serum, whole blood, and saliva using novel plasmonic gold (pGOLD) chips with promising results [6]. However, these two approaches are not yet widely used in the conventional diagnosis of CT. Postnatal screening and follow-up of neonates are essentially based on serological tests: detection of specific IgM and/or IgA using immunoanalysis, monitoring IgG kinetics during follow-up using immunoanalysis, and detecting supplemental IgG and/or IgM anti-Toxoplasma in the newborn using immunoblot. Conventionally, supplementary band(s) in newborn patterns indicate specific antibody neosynthesis in the newborn’s serum, also confirming the CT diagnosis. The sensitivity of IgG and IgM immunoblot at birth (cord blood or J3 serum) is 65% to 79% [10], reaching as high as 95.8% in the combination of WB IgM with prenatal and serological neonatal tests during the first month of life [3]. Immunologic profile testing makes it possible to determine when individualized antibodies are synthesized by the newborn following toxoplasmosis infection. CT remains a continued challenge for pregnancy and due to the severe potential sequelae, it is still necessary to improve timely neonatal diagnosis. Our findings propose to supplement conventional reading of the immunoblot with the presence of the three IgM bands in the infant pattern: 75, 90 and 100 kDa. The IgM triplet appears to be pathognomonic for the diagnosis of CT. No IgM triplet was observed in the group of uninfected infants (*n* = 237). This enables us to increase sensitivity of the immunoblot assay appreciably from 55.0% to 72.5%. It is emphasized that the new reading comprises both the conventional reading and the presence of the infant’s IgM triplet, regardless of whether it is present or not in the mother’s pattern. This is the first time that a test has shown a specific marker of infection in the newborn at birth, using different immunologic patterns. For the IgM triplet, a different profile showing antibody neosynthesis should not be sought. This is a new concept because these three IgM bands do not reflect neosynthesis, but probably an immunologic response against proteins involved in mother-to-child transmission. We also emphasize that this new interpretation must be integrated into the overall approach to CT diagnosis and in no way replaces the IgM/IgA immunoanalysis assay or the monitoring of IgG kinetics during follow-up. The preferential occurrence of the IgM triplet when the time of infection is the third trimester is interesting because it is sometimes too late to program prenatal diagnosis at this term. The IgM triplet can then make up for this lack of information. A proteomics analysis should be undertaken to identify these specific proteins. Most of the studies on the pathogenesis of vertical *T. gondii* focus on the immune response to parasite antigen stimulation, but few data describe the proteins involved in transplacental invasion [1]. In fact, *T. gondii* is one of the few pathogens that can cross the placenta, which probably involves a strict specific invasive process. Moreover, based on French National Reference Center data, most CT diagnoses are made on post-natal diagnosis, which highlights the utility of the triplet [15]. The IgM triplet may be one of the avenues to explore to understand placental responses to *T. gondii* infection. Finally, a larger study could be performed among diagnostic laboratories in association with the NRCT to extend these data.

## Supplementary information

The supplementary material of this article is available at https://www.parasite-journal.org/10.1051/parasite/2023020/olm. *Table S1*. Detail of the retrospective reading of the immunoblot mother-child pairs in the CT group. POS: positive result; NEG: negative result; trim: trimester; ND: no data; NP: not performed.
