# Supplementary material for: IgM triplet in neonatal diagnosis by immunoblotting and its potential use as a diagnostic marker for congenital toxoplasmosis
Source: Parasite. 2023 Jun 2;30:19. doi: 10.1051/parasite/2023020 (PMC10236937; doi:10.1051/parasite/2023020)
Supplement: Supplementary file 1 — Table S1. Detail of the retrospective reading of the immunoblot mother-child pairs in the CT group. POS: positive result; NEG: negative result; trim: trimester; ND: no data; NP: not performed. [file parasite-30-19-s1.pdf]

Patient	Trimester of maternal infection	Amniotic fluid qPCR results	IgM triplet of the mother	IgM triplet of the infant	Conventional reading IgG and IgM profiles (1: different pattern; 0: no supplementary bands)	Conventional reading + presence of the infant's IgM triplet (1: CT diagnosis, 0: absence of arguments for CT diagnosis by IB)	ISAgA or Platelia Toxo IgM
1	1st	NP	YES	YES	1	1	POS
2	1st	NP	YES	YES	0	1	POS
3	1st	NEG	NO	NO	0	0	NEG
4	1st	NP	YES	YES	0	1	POS
5	1st	POS	NO	NO	0	0	NEG
6	1st	NEG	NO	NO	0	0	POS
7	1st	NP	NO	NO	0	0	NEG
8	1st	NEG	YES	YES	1	1	POS
9	1st	NP	NO	NO	1	1	NEG
10	1st	NEG	YES	YES	1	1	POS
11	1st	POS	YES	YES	1	1	POS
12	1st	POS	NO	YES	1	1	NEG
13	1st	POS	YES	NO	0	0	NEG
14	1st	NEG	NO	NO	1	1	POS
15	1st	POS	NO	NO	1	1	POS
16	1st	POS	NO	NO	1	1	NEG
17	2nd	POS	NO	NO	0	0	NEG
18	2nd	POS	NO	NO	0	0	POS
19	2nd	POS	NO	NO	0	0	NEG
20	2nd	POS	YES	NO	0	0	POS
21	2nd	POS	NO	NO	0	0	NEG
22	2nd	POS	NO	NO	0	0	NEG
23	2nd	POS	NO	NO	0	0	NEG
24	2nd	POS	YES	YES	0	1	NR
25	2nd	POS	NO	NO	0	0	NEG
26	2nd	POS	NO	NO	0	0	NEG
27	2nd	NP	NO	NO	1	1	POS
28	2nd	POS	YES	YES	1	1	POS
29	2nd	POS	NO	NO	1	1	NEG
30	2nd	POS	NO	NO	0	0	NEG
31	2nd	POS	NO	NO	0	0	NEG
32	2nd	POS	YES	NO	0	0	POS
33	2nd	NP	NO	YES	1	1	POS
34	2nd	NP	NO	YES	1	1	POS
35	2nd	POS	NO	YES	1	1	NEG
36	2nd	POS	NO	NO	0	0	NEG
37	2nd	POS	NO	NO	0	0	NEG

38	2nd	POS	NO	NO	1	1	NEG
39	2nd	NEG	NO	NO	1	1	POS
40	2nd	POS	YES	YES	1	1	POS
41	2nd	POS	YES	NO	0	0	NEG
42	2nd	POS	YES	NO	0	0	POS
43	2nd	POS	YES	YES	0	1	POS
44	2nd	NP	YES	YES	0	1	POS
45	2nd	POS	NO	NO	1	1	NEG
46	2nd	POS	YES	YES	1	1	POS
47	2nd	POS	YES	YES	0	1	NEG
48	2nd	POS	NO	NO	1	1	NEG
49	2nd	POS	YES	NO	0	0	NEG
50	2nd	POS	NO	NO	0	0	POS
51	2nd	POS	NO	NO	0	0	NEG
52	2nd	POS	NO	NO	1	1	NEG
53	2nd	NP	NO	NO	1	1	POS
54	2nd	POS	NO	YES	1	1	POS
55	2nd	POS	YES	YES	0	1	POS
56	2nd	NP	NO	YES	0	1	NEG
57	2nd	POS	YES	YES	1	1	POS
58	2nd	POS	NO	YES	0	1	NEG
59	2nd	POS	YES	YES	0	1	NEG
60	2nd	NP	YES	YES	0	1	NEG
61	2nd	POS	NO	NO	0	0	POS
62	2nd	NP	NO	NO	0	0	POS
63	2nd	POS	NO	NO	1	1	POS
64	2nd	NP	NO	NO	0	0	POS
65	2nd	POS	YES	YES	1	1	POS
66	2nd	POS	NO	YES	1	1	NEG
67	2nd	POS	YES	YES	1	1	POS
68	2nd	POS	YES	YES	0	1	POS
69	2nd	POS	NO	NO	0	0	NEG
70	2nd	POS	NO	NO	0	0	NEG
71	2nd	NP	NO	NO	1	1	POS
72	2nd	POS	NO	NO	1	1	POS
73	2nd	POS	YES	YES	1	1	POS
74	2nd	NP	NO	YES	1	1	POS
75	2nd	POS	YES	YES	1	1	NEG
76	2nd	NP	YES	YES	1	1	NEG
77	2nd	POS	YES	NO	0	0	NEG
78	2nd	NEG	YES	NO	0	0	NEG
79	2nd	POS	NO	NO	0	0	NEG
80	2nd	NEG	NO	NO	0	0	POS
81	2nd	POS	NO	NO	0	0	POS
82	2nd	POS	NO	NO	1	1	POS

83	2nd	POS	YES	YES	1	1	POS
84	2nd	NP	YES	YES	0	1	POS
85	2nd	POS	YES	YES	0	1	POS
86	2nd	POS	NO	NO	0	0	POS
87	2nd	POS	NO	NO	1	1	POS
88	2nd	NP	NO	NO	1	1	POS
89	2nd	NP	NO	NO	0	0	POS
90	2nd	NEG	NO	NO	0	0	NEG
91	3rd	NP	YES	YES	0	1	POS
92	3rd	ND	YES	YES	1	1	POS
93	3rd	POS	NO	YES	1	1	POS
94	3rd	POS	YES	YES	0	1	NEG
95	3rd	POS	NO	NO	1	1	POS
96	3rd	POS	NO	NO	0	0	NEG
97	3rd	POS	YES	YES	1	1	POS
98	3rd	POS	NO	YES	1	1	POS
99	3rd	POS	NO	YES	1	1	POS
100	3rd	POS	YES	YES	0	1	NEG
101	3rd	NP	NO	YES	0	1	POS
102	3rd	POS	YES	YES	0	1	NEG
103	3rd	NP	YES	YES	0	1	POS
104	3rd	POS	NO	NO	0	0	POS
105	3rd	POS	NO	NO	0	0	POS
106	3rd	ND	NO	NO	0	0	NEG
107	3rd	POS	NO	NO	0	0	POS
108	3rd	NP	NO	NO	0	0	NEG
109	3rd	NP	NO	NO	0	0	NEG
110	3rd	POS	NO	NO	0	0	POS
111	3rd	POS	NO	NO	0	0	NEG
112	3rd	POS	NO	NO	1	1	POS
113	3rd	POS	NO	NO	0	0	POS
114	3rd	NP	NO	NO	1	1	POS
115	3rd	ND	NO	YES	1	1	POS
116	3rd	POS	YES	YES	1	1	POS
117	3rd	ND	YES	YES	1	1	POS
118	3rd	ND	YES	YES	1	1	POS
119	3rd	POS	YES	YES	1	1	POS
120	3rd	NP	YES	YES	1	1	POS
121	3rd	POS	NO	NO	0	0	NEG
122	3rd	NP	NO	NO	1	1	POS
123	3rd	ND	NO	YES	1	1	POS
124	3rd	POS	NO	YES	0	1	POS
125	3rd	POS	YES	YES	0	1	POS
126	3rd	NP	NO	NO	0	0	NEG
127	3rd	POS	NO	YES	1	1	NEG

128	3rd	ND	YES	YES	1	1	POS
129	3rd	POS	NO	YES	1	1	POS
130	3rd	NP	YES	YES	1	1	POS
131	3rd	NP	YES	YES	1	1	POS
132	3rd	NP	YES	YES	1	1	POS
133	3rd	ND	YES	YES	1	1	POS
134	3rd	ND	YES	YES	0	1	POS
135	3rd	NP	NO	NO	0	0	POS
136	3rd	POS	YES	YES	1	1	POS
137	3rd	NP	YES	YES	1	1	POS
138	3rd	ND	YES	YES	1	1	POS
139	3rd	POS	NO	YES	1	1	POS
140	3rd	NP	YES	YES	1	1	POS
141	3rd	NP	YES	YES	1	1	POS
142	3rd	ND	YES	NO	0	0	POS
143	3rd	NP	YES	YES	1	1	POS
144	3rd	ND	NO	YES	1	1	POS
145	3rd	NP	YES	YES	1	1	POS
146	3rd	NP	YES	YES	1	1	POS
147	3rd	NP	YES	YES	0	1	POS
148	3rd	POS	NO	NO	0	0	POS
149	3rd	NP	NO	NO	0	0	NEG
150	3rd	ND	NO	NO	1	1	POS
151	3rd	NP	YES	NO	0	0	NEG
152	3rd	NP	NO	NO	0	0	NEG
153	3rd	NP	NO	NO	1	1	POS
154	3rd	NP	NO	YES	1	1	POS
155	3rd	ND	NO	YES	1	1	POS
156	3rd	POS	YES	YES	1	1	POS
157	3rd	POS	NO	YES	1	1	POS
158	3rd	NP	YES	YES	1	1	POS
159	3rd	NP	NO	YES	1	1	POS
160	3rd	NP	NO	YES	1	1	POS
161	3rd	NP	YES	YES	0	1	POS
162	3rd	NP	YES	YES	0	1	POS
163	3rd	NP	NO	YES	0	1	POS
164	3rd	NP	YES	YES	0	1	POS
165	3rd	NP	YES	YES	0	1	POS
166	3rd	NP	YES	YES	0	1	POS
167	3rd	POS	YES	YES	0	1	NR
168	3rd	ND	NO	NO	1	1	POS
169	3rd	POS	NO	NO	0	0	POS
170	3rd	NP	NO	NO	0	0	POS
171	3rd	NP	NO	NO	1	1	POS
172	3rd	NP	NO	YES	1	1	POS

173	3rd	ND	NO	YES	1	1	POS
174	3rd	ND	YES	YES	1	1	POS
175	3rd	NP	YES	YES	0	1	POS
176	3rd	POS	NO	YES	0	1	NEG
177	3rd	NP	YES	YES	0	1	POS
178	3rd	NP	NO	NO	0	0	POS
179	3rd	NP	NO	NO	1	1	POS
180	3rd	NP	NO	NO	0	0	NEG
181	3rd	NP	NO	YES	1	1	POS
182	3rd	NP	NO	YES	0	1	POS
183	3rd	NP	NO	YES	0	1	POS
184	3rd	NP	NO	YES	0	1	POS
185	3rd	NP	NO	NO	0	0	NEG
186	3rd	POS	NO	NO	1	1	POS
187	3rd	NP	NO	NO	0	0	NEG
188	3rd	NP	NO	NO	0	0	NEG
189	3rd	NP	NO	NO	1	1	POS
190	3rd	NP	YES	YES	1	1	NEG
191	3rd	NP	NO	YES	1	1	POS
192	3rd	POS	NO	YES	1	1	POS
193	3rd	NP	NO	YES	1	1	POS
194	3rd	NP	YES	YES	1	1	POS
195	3rd	POS	NO	NO	0	0	NEG
196	3rd	NP	YES	YES	0	1	POS
197	3rd	NP	YES	YES	0	1	POS
198	3rd	NP	NO	YES	0	1	POS
199	3rd	NP	NO	YES	0	1	NEG
200	3rd	NP	NO	NO	0	0	POS
201	3rd	NP	NO	YES	1	1	POS
202	3rd	NP	NO	NO	0	0	POS
203	3rd	NP	NO	YES	1	1	POS
204	3rd	NEG	YES	YES	1	1	POS
205	3rd	POS	YES	YES	1	1	POS
206	3rd	ND	YES	YES	1	1	POS
207	3rd	ND	NO	YES	1	1	POS
208	3rd	NEG	NO	NO	1	1	POS
209	3rd	ND	YES	YES	1	1	POS
210	3rd	ND	YES	NO	1	1	POS
211	3rd	NP	NO	NO	1	1	POS
212	3rd	POS	YES	YES	1	1	POS
213	3rd	ND	NO	YES	1	1	POS
214	3rd	POS	NO	YES	1	1	POS
215	3rd	ND	NO	YES	1	1	POS
216	3rd	ND	NO	YES	1	1	POS
217	3rd	ND	YES	YES	1	1	POS

218	3rd	ND	YES	YES	1	1	POS
219	3rd	ND	YES	YES	1	1	POS
220	3rd	ND	YES	YES	1	1	POS
221	3rd	NP	YES	YES	1	1	POS
222	3rd	POS	NO	NO	0	0	NEG
223	3rd	ND	NO	YES	0	1	NEG
224	ND	NP	NO	NO	1	1	POS
225	ND	POS	NO	NO	1	1	POS
226	ND	ND	NO	NO	1	1	POS
227	ND	ND	NO	NO	1	1	POS
228	ND	NEG	NO	NO	0	0	NEG
229	ND	POS	NO	NO	1	1	POS
230	ND	NEG	NO	NO	0	0	NEG
231	ND	ND	NO	YES	1	1	POS
232	ND	POS	NO	YES	1	1	POS
233	ND	NP	NO	NO	1	1	POS
234	ND	ND	YES	YES	1	1	POS
235	ND	NP	NO	NO	1	1	POS
236	ND	NP	NO	NO	1	1	POS
237	ND	ND	YES	YES	1	1	POS
238	ND	POS	NO	NO	1	1	POS
239	ND	ND	NO	YES	1	1	POS
240	ND	POS	NO	NO	0	0	NEG
241	ND	ND	NO	YES	1	1	POS
242	ND	NP	NO	YES	1	1	POS
243	ND	NP	NO	NO	0	0	NEG
244	ND	ND	NO	YES	1	1	POS
245	ND	POS	NO	YES	1	1	POS
246	ND	NP	NO	YES	1	1	POS
247	ND	NP	YES	YES	1	1	POS
248	ND	ND	NO	YES	1	1	POS
249	ND	NP	YES	NO	0	0	POS
250	ND	NP	NO	NO	1	1	POS
251	ND	NP	YES	NO	1	1	POS
252	ND	NP	NO	NO	1	1	POS
253	ND	NP	NO	NO	1	1	NEG
254	ND	POS	NO	NO	1	1	POS
255	ND	POS	YES	YES	0	1	NEG
256	ND	NP	NO	NO	1	1	NEG
257	ND	POS	NO	YES	0	1	NEG
258	ND	POS	YES	YES	0	1	POS

Table 2 (Supplementary information). Detail of the retrospective reading of the immunoblot mother-child pair in the CT group. POS: positive result; NEG: negative result; trim: trimester; ND: no data; NP: not performed.
